# Supplementary material for: T6SS Accessory Proteins, Including DUF2169 Domain-Containing Protein and Pentapeptide Repeats Protein, Contribute to Bacterial Virulence in T6SS Group_5 of Burkholderia glumae BGR1
Source: Plants (Basel). 2021 Dec 23;11(1):34. doi: 10.3390/plants11010034 (PMC8747328; doi:10.3390/plants11010034)
Supplement: Supplementary file 1 [file plants-11-00034-s001.zip › plants-1499155-supplementary.pdf]

Supplementary Table S1. Analysis of deletion site in *Δu1g23320*.

Strain	Sequence (5`→3`)
Wild type BGR1	ATGTGGCGCGTCGATAACGGGACCCCGTTTGTTGCAGATCGGACTTGGGT
Δu1g23320	ATGTGGCGCGTCGAT- -
Wild type BGR1	GCGAGATTCCGACGGAGCGGAAGTCTGGGTGGTCGCCGTGAAGGCAACCT
Δu1g23320	- -
Wild type BGR1	ATAGCATTCTGCCAGACGGAAACATCCGGCTCGCCGGTGAACAGCGACCC
Δu1g23320	- -
Wild type BGR1	TTGAATCACGGGCCGGTACCGGCCGACGGGCGTGCGGGCTTGCTCTACGA
Δu1g23320	- -
Wild type BGR1	CACCGATCTCGGGCCACCCAAGACGGCGACCGACATCGTCCTGAACGGCT
Δu1g23320	- -
Wild type BGR1	CTGCGTGGGCACCGAATGGGGAGCCGGTGAGGAGGCTGCGGGCGGGATTT
Δu1g23320	- -
Wild type BGR1	CAGATTGGCGCGACTGCCCACTTTGCCTTGGTGTGGGGCGATCGGTATTG
Δu1g23320	- -
Wild type BGR1	GGAGCGCAAGCTCTGGTCGTGGCGACCCAGTAAGGCCGTGCCTTTCGTGA
Δu1g23320	- -
Wild type BGR1	GCATGCCGCTCGCCTATGAGCACGCATTCGGCGGTGATACGCCGGAATTA
Δu1g23320	- -
Wild type BGR1	CCGCCGGCGAGCCGTAACCCTGTTGGGCGAGGTTATGCGCCCGACGCTGA
Δu1g23320	- -
Wild type BGR1	TGGACGCGTCTGGTTGCCAAATATTGAATCCGTGACGAAGCCGATTCGCA
Δu1g23320	- -
Wild type BGR1	AGCGAGACGACGTGCCGCCAACTACGGGGTTCGGTGTACTTCCCGCTCAT
Δu1g23320	- -
Wild type BGR1	TGGCCAGCGAGGCTGCAGCATGCGGGCACGTACGACGACACTTGGCGTAA
Δu1g23320	- -
Wild type BGR1	CACACGACACCCCCTACCACCCGAGGACCTGGACCCTCGCTTCTGGCAGA
Δu1g23320	- -
Wild type BGR1	TCGCGCCGCCCGAGCAGCAAACGAACCGCCACTTGTCGGGCGGCGAAACG
Δu1g23320	- -
Wild type BGR1	GTGACGCTCCTCAATGTGACACGGCCGGGGTGGGTGCCCGGCGGCCGCTT
Δu1g23320	- -
Wild type BGR1	GAACTTCCGCTTGCCCAAGGTTTCGCTGGTGTGTGAGACGCGCTTTTACG
Δu1g23320	- -
Wild type BGR1	ACGGCACACGGGTGCACACCCGGCCGGTGATACACACGGTCATCCTCGAG
Δu1g23320	- -
Wild type BGR1	CCGGACGAGCTTCGCGTATGCGTGGTGTACCACATGGCGTTGCCCTGTCA
Δu1g23320	- -
Wild type BGR1	CGAGAAGGTCAATTTGCTGGACCAGACCTGCGTGACGATGAAGCGTCGAC
Δu1g23320	------------------------------CTGGACCAGACCTGCGTGACGATGAAGCGTCGAC
Wild type BGR1	CACTCGATAAGGCGTGGCAAGCCGTGTTCGGCGATGTCCTGACCGCAGCC
Δu1g23320	CACTCGATAAGGCGTGGCAAGCCGTGTTCGGCGATGTCCTGACCGCAGCC
Wild type BGR1	GAGGCGAGCCCTGAATCCGACACGGGGCAATCCGCATGA
Δu1g23320	GAGGCGAGCCCTGAATCCGACACGGGGCAATCCGCATGA

Supplementary Table S2. Analysis of deletion site in *Δu2g07410*.

Strain	Sequence (5`→3`)
Wild type BGR1	ATGAGCACGATCTGGGACGACATCGCGCTGGCGATCCAGCTCGACCAGCC
Δu2g07410	ATGAGCACGATCTGGGACGACATCGCGCTGGCGATCCAGCTCGACCAGCC
Wild type BGR1	GATCAGGCAGCGCGAGCTGGCCGGGCTCGACGCGCGCCGCCGCGAGCTGG
Δu2g07410	GATCAGGCAGCGCGAGCTGGCCGGGCTCGACGCGCGCCGCCGCGAGCTGG
Wild type BGR1	GCGGCGCGGCGTTCAGCGAGGTGGACTTCTCGCACGCCTCGTTCGCCGAC
Δu2g07410	GCGGCGCGGCGTTCAGCGAGGTGGACTTCTC-----------------------------------
Wild type BGR1	GCCTCGCTGGCGCGCGCGCTTCATCGAGTGCGACCTCAGCGACGCCGA
Δu2g07410	- -
Wild type BGR1	CTTCGGCAACGCCGATCTCGACCACGCGAGCTTCGTCAAGTGCAAGCTGC
Δu2g07410	- -
Wild type BGR1	CGCGCGCGCGGCTCGCCGGCCGCAACATGAAGCACGCCTGCTGGGTGGAT
Δu2g07410	- -
Wild type BGR1	TGCGACCTCAGCGACAGCGACTGGAGCCACGTGCAGATGCAGACCAGCTC
Δu2g07410	- -
Wild type BGR1	GATCGTCAACAGCGCGCTCGAGGGCGCGCGCTTCGCGGCCGGCACGCTCA
Δu2g07410	- -
Wild type BGR1	CGCGCTGCACGCTGATCCAGGACGGCTTCGAGGGCGCCAGCGTGGCCGGC
Δu2g07410	- -
Wild type BGR1	GCGACGCTCGAGCGCTGCGTGGTGAGCCCGGCCGACTGGCGCGAGGTGGA
Δu2g07410	- -
Wild type BGR1	CCTGCGCGGGCTGAGCGCCACCGGGGTGGTGTTCTCGAAGGCGAACTTCA
Δu2g07410	- -
Wild type BGR1	GCGGCCAGCTCATGAGCGGCCTGCGCCTGCGGCGCTGCTCGCTGCAGGCA
Δu2g07410	- -
Wild type BGR1	GCGGAGCTCGCCCAGGTCGATCTGGCGGGCGCCGACCTGACCCAGTCGAA
Δu2g07410	- -
Wild type BGR1	CCTGTACCAGGCGCGCCTCACGCAGGCCAACCTCAGCAACGTCGAGGCCG
Δu2g07410	- -
Wild type BGR1	GCCACGCGCTGTTCTATCAGGCGAGCCTCGACCACGTGAACCTGGCGGAG
Δu2g07410	- -
Wild type BGR1	GCCAGGCTCGACGGCGCGATCTTCCAGCAGGCGAGCCTGGCCGACTGCCG
Δu2g07410	- -
Wild type BGR1	GCTCGACGGCGCGCACCTGAAGGCCAGCATCTGGAACAAGAGCCACCACC
Δu2g07410	- -
Wild type BGR1	AGCGCGTGTCGTTCCGCGGCGCCGAACTCGTCCACGCGCGCCTCGAACAC
Δu2g07410	- -
Wild type BGR1	AATGCATAACGCGACGCTGTGCGACGCGCGGTTTCGCGGAGCCAGCCTGC
Δu2g07410	---------ATAACGCGACGCTGTGCGACGCGCGGTTTCGCGGAGCCAGCCTGC
Wild type BGR1	GCCAGGTCGGCCGGACCGATCCGGCGCGCCTCTCGGCCGAAGCCCGTACC
Δu2g07410	GCCAGGTCGGCCGGACCGATCCGGCGCGCCTCTCGGCCGAAGCCCGTACC
Wild type BGR1	CTTGCCCTGGAGAACGAATGA
Δu2g07410	CTTGCCCTGGAGAACGAATGA

Supplementary Table S3. Analysis of deletion site in *Δu2g07420DUF2169* and *Δu2g07420PPR*.

Strain	Sequence (5`→3`)
Wild type BGR1	ATGAAAATCATCAAACCCCAATCGCTCGGCCTGCTGCAGAAGCCCTACAC
Δu2g07420DUF2169	ATGAAAATCATCAAACCCCAATCGCTCGGCCTGCTGCAGAAGCCCTACAC
Δu2g07420PPR	ATGAAAATCATCAAACCCCAATCGCTCGGCCTGCTGCAGAAGCCCTACAC
Wild type BGR1	CCACCTCGGCCGGCATCGGCTGTCGGTGGCCGTGATCGGCTTCTTCCCGC
Δu2g07420DUF2169	CCACCTCGGCCGGCATCGGCTGTCGGTGGCCGTGATCGGCTTCTTCCCGC
Δu2g07420PPR	CCACCTCGGCCGGCATCGGCTGTCGGTGGCCGTGATCGGCTTCTTCCCGC
Wild type BGR1	TCGGCCGGACCAGCGAGCGCTTCCTCGCCGAGAACCAGCAATGGCCGCAC
Δu2g07420DUF2169	TCGGCCGGACCAGCGAGCGCTTCCTCGCCGAGAACCAGCAATGGCCG------
Δu2g07420PPR	TCGGCCGGACCAGCGAGCGCTTCCTCGCCGAGAACCAGCAATGGCCGCAC
Wild type BGR1	GTGCTGGCGAGCCTGCCGGCCGGCCAGCCGCTCGACGAGGCGATGCCGCG
Δu2g07420DUF2169	- -
Δu2g07420PPR	GTGCTGGCGAGCCTGCCGGCCGGCCAGCCGCTCGACGAGGCGATGCCGCG
Wild type BGR1	CCAGGGCGCCGAGGTGCTGCTGCTCGGCTCGGCCTACGCGCCGCAGCGCA
Δu2g07420DUF2169	- -
Δu2g07420PPR	CCAGGGCGCCGAGGTGCTGCTGCTCGGCTCGGCCTACGCGCCGCAGCGCA
Wild type BGR1	AGGCCGCCACCAGCGTGGACGTGCAGCTGCGGCTCGACGACCGCGCCGGC
Δu2g07420DUF2169	- -
Δu2g07420PPR	AGGCCGCCACCAGCGTGGACGTGCAGCTGCGGCTCGACGACCGCGCCGGC
Wild type BGR1	CAGCCGCTCGTCAGCAAGTGCCTGTGCGTGTGCGGCGAGCGCGAATGGCG
Δu2g07420DUF2169	- -
Δu2g07420PPR	CAGCCGCTCGTCAGCAAGTGCCTGTGCGTGTGCGGCGAGCGCGAATGGCG
Wild type BGR1	CGCGCCGGCGCTGGGCGGGCGCCGCGTCGGCCAGCCCAAGCCGTTCCTCG
Δu2g07420DUF2169	- -
Δu2g07420PPR	CGCGCCGGCGCTGGGCGGGCGCCGCGTCGGCCAGCCCAAGCCGTTCCTCG
Wild type BGR1	CGATGCCGCTCAGCTATGCGCGCGCGTTCGGCGGCGCGCGCCACGCGGCC
Δu2g07420DUF2169	- -
Δu2g07420PPR	CGATGCCGCTCAGCTATGCGCGCGCGTTCGGCGGCGCGCGCCACGCGGCC
Wild type BGR1	AACCCGGCCGGCTGCGGCAGCCGTGCGTCGTGGTTCGGCAAGGGCCGCGG
Δu2g07420DUF2169	- -
Δu2g07420PPR	AACCCGGCCGGCTGCGGCAGCCGTGCGTCGTGGTTCGGCAAGGGCCGCGG
Wild type BGR1	CGCGATGCCGAACGTCGCCTACCCGCCCAACGCGGCGGATCCCGCCTGGC
Δu2g07420DUF2169	- -
Δu2g07420PPR	CGCGATGCCGAACGTCGCCTACCCGCCCAACGCGGCGGATCCCGCCTGGC
Wild type BGR1	GCGCCAGCGTGCCGGCCGGCTTCGGGCCGATCCCGATCGGCAACCGCGCG
Δu2g07420DUF2169	- -
Δu2g07420PPR	GCGCCAGCGTGCCGGCCGGCTTCGGGCCGATCCCGATCGGCAACCGCGCG
Wild type BGR1	CGGCTGCGCAAGTTCGGCAGCTACGGCAGCCAGTGGCTCAAGCACGACGC
Δu2g07420DUF2169	- -
Δu2g07420PPR	CGGCTGCGCAAGTTCGGCAGCTACGGCAGCCAGTGGCTCAAGCACGACGC
Wild type BGR1	GCCCGGCTTCGCGCGCGACCTCGACTGGAGCGTGTTCAACATGGCGCCGC
Δu2g07420DUF2169	- -
Δu2g07420PPR	GCCCGGCTTCGCGCGCGACCTCGACTGGAGCGTGTTCAACATGGCGCCGC
Wild type BGR1	CCGACCAGTGGACGAAGGCGCCGTTCCAGGGCGGCGAGCGCTACACGCTG
Δu2g07420DUF2169	- -
Δu2g07420PPR	CCGACCAGTGGACGAAGGCGCCGTTCCAGGGCGGCGAGCGCTACACGCTG
Wild type BGR1	CGGCATCTGCATCCGCAGCACGCCGAGCTGTCCGGCACCCTGCCCGGGCT
Δu2g07420DUF2169	- -
Δu2g07420PPR	CGGCATCTGCATCCGCAGCACGCCGAGCTGTCCGGCACCCTGCCCGGGCT
Wild type BGR1	GGCCGCGCGCGGCTTCGTGCTGCGCGCCGGCGAGGCGCCCGAGCAGGCCC
Δu2g07420DUF2169	- -
Δu2g07420PPR	GGCCGCGCGCGGCTTCGTGCTGCGCGCCGGCGAGGCGCCCGAGCAGGCCC
Wild type BGR1	CTCGGCGTCGCGGTCTATCACGGCACGCTCGAGATCGGCGACGGCGACGG
Δu2g07420DUF2169	- -
Δu2g07420PPR	CTCGGCGTCGCGGTCTATCACGGCACGCTCGAGATCGGCGACGGCGACGG
Wild type BGR1	GCTCGACGTCGACACGCTGATGGTCGGCTACGAACACCGCGACCGCCCGA
Δu2g07420DUF2169	- -

Δu2g07420PPR	GCTCGACGTCGACACGCTGATGGTCGGCTACGAACACCGCGACCGCCCGA
Wild type BGR1	AGCCCCTGGCGCACTACCACGACGTGCTGCGGCTGCGCCTCGACCCCGAG
Δu2g07420DUF2169	- -
Δu2g07420PPR	AGCCCCTGGCGCACTACCACGACGTGCTGCGGCTGCGCCTCGACCCCGAG
Wild type BGR1	GCGGCCCGCCTGCACGCGTTCAACGACAGCCAGCTCGCGCCGCAGCGTTC
Δu2g07420DUF2169	- -
Δu2g07420PPR	GCGGCCCGCCTGCACGCGTTCAACGACAGCCAGCTCGCGCCGCAGCGTTC
Wild type BGR1	CGAGGCCGAGCAGGCGCGCCGCGCCCGCGAGCAGCAAGCGGCCGAGCAGG
Δu2g07420DUF2169	- -
Δu2g07420PPR	CGAGGCCGAGCAGGCGCGCCGCGCCCGCGAGCAGCAAGCGGCCGAGCAGG
Wild type BGR1	CCGTGCTGGCGCGCGACCAGCGCCGCCTCGACCTGCTCGACCAGCAATAC
Δu2g07420DUF2169	- -
Δu2g07420PPR	CCGTGCTGGCGCGCGACCAGCGCCGCCTCGACCTGCTCGACCAGCAATAC
Wild type BGR1	TGGGCGCGCCGCGGCAAGGCCCGGCCGGCCGGCCATCAGGTCGCGCAAGC
Δu2g07420DUF2169	- -
Δu2g07420PPR	TGGGCGCGCCGCGGCAAGGCCCGGCCGGCCGGCCATCAGGTCGCGCAAGC
Wild type BGR1	CCGGCTGCCGGCGCTCGGCCTGATGACCTCGCAGACGGCGGCCGAGGGCG
Δu2g07420DUF2169	- -
Δu2g07420PPR	CCGGCTGCCGGCGCTCGGCCTGATGACCTCGCAGACGGCGGCCGAGGGCG
Wild type BGR1	ACTTCGATCTGTCGGAGATCGTGCTGAAGGCCAAGGCGCTGGCGGCCGAC
Δu2g07420DUF2169	- -
Δu2g07420PPR	ACTTCGATCTGTCGGAGATCGTGCTGAAGGCCAAGGCGCTGGCGGCCGAC
Wild type BGR1	GCCGAGCAGCGCGGCCTGGCGGCGCTCGCCAAGCTGCCGGCCCGGCCGGC
Δu2g07420DUF2169	- -
Δu2g07420PPR	GCCGAGCAGCGCGGCCTGGCGGCGCTCGCCAAGCTGCCGGCCCGGCCGGC
Wild type BGR1	GGCGGCGGTGGACGCGGCCAAGCTGCTGGCCGACGCGCTCGAACGCGCGG
Δu2g07420DUF2169	- -
Δu2g07420PPR	GGCGGCGGTGGACGCGGCCAAGCTGCTGGCCGACGCGCTCGAACGCGCGG
Wild type BGR1	CGCGGCCGGCCTACGACCTGCTCGCGCCGGAGCAGGCCGGGCGCGATCCG
Δu2g07420DUF2169	- -
Δu2g07420PPR	CGCGGCCGGCCTACGACCTGCTCGCGCCGGAGCAGGCCGGGCGCGATCCG
Wild type BGR1	CAGGTCGCCAGCATGCTGGCCAAGCTCGCCGCGCCGGCCGCCGACGCCGG
Δu2g07420DUF2169	- -
Δu2g07420PPR	CAGGTCGCCAGCATGCTGGCCAAGCTCGCCGCGCCGGCCGCCGACGCCGG
Wild type BGR1	CCCGAAGCAGCACGAGCGCCACCAGCACGCGCGCGACGCGGTCCTGAAGA
Δu2g07420DUF2169	- -
Δu2g07420PPR	CCCGAAGCAGCACGAGCGCCACCAGCACGCGCGCGACGCGGTCCTGAAGA
Wild type BGR1	TTCCGGCGCTGCGGCGCCAGGCGCGCCGCTCGGCGCCGAAGCCGACGCTC
Δu2g07420DUF2169	------------------------GCGCCAGGCGCGCCGCTCGGCGCCGAAGCCGACGCTC
Δu2g07420PPR	TTCCGGCGCTGCGGCGCCAGGCGCGCCGCTCGGCGCCGAAGCCGACGCTC
Wild type BGR1	GCCGCGCTGCCCTATCCGGCCGACGTCGCGCGCCGCCTCGGCGAGCAGAT
Δu2g07420DUF2169	GCCGCGCTGCCCTATCCGGCCGACGTCGCGCGCCGCCTCGGCGAGCAGAT
Δu2g07420PPR	GCCGCGCTGCCCTATCCGGCCGACGTCGCGCGCCGCCTCGGCGAGCAGAT
Wild type BGR1	CCGCCAATGGCACGCGGCGGGCGTGGTGCTGGCCGGGCGCGACCTGGCCG
Δu2g07420DUF2169	CCGCCAATGGCACGCGGCGGGCGTGGTGCTGGCCGGGCGCGACCTGGCCG
Δu2g07420PPR	CCGCCA--
Wild type BGR1	GCGCCGATCTGGCCGGCCTCGATTTCTCGGGCGCCGACCTGCGCGAGGCG
Δu2g07420DUF2169	GCGCCGATCTGGCCGGCCTCGATTTCTCGGGCGCCGACCTGCGCGAGGCG
Δu2g07420PPR	- -
Wild type BGR1	ATGCTCGACGGCGCCGACCTCAGCGGGGCGAAATTCGTCGGCGCCAAGCT
Δu2g07420DUF2169	ATGCTCGACGGCGCCGACCTCAGCGGGGCGAAATTCGTCGGCGCCAAGCT
Δu2g07420PPR	- -
Wild type BGR1	GCAGGGCGCCGTGCTGGTGGGCGCGCGCCTGGACCACGCCGATTTTTCGG
Δu2g07420DUF2169	GCAGGGCGCCGTGCTGGTGGGCGCGCGCCTGGACCACGCCGATTTTTCGG
Δu2g07420PPR	- -
Wild type BGR1	GCGCCGACCTCACGCGCGCCAACCTCTGCATGAGCAGCGGCCGCGCCATC
Δu2g07420DUF2169	GCGCCGACCTCACGCGCGCCAACCTCTGCATGAGCAGCGGCCGCGCCATC
Δu2g07420PPR	- -

Wild type BGR1<br>*Δu2g07420DUF2169*<br>*Δu2g07420PPR*	TCGTTCGACGGCGCCGAGCTGGGCCACGCCCAGGCGATCGACGCGCAGTG<br>TCGTTCGACGGCGCCGAGCTGGGCCACGCCCAGGCGATCGACGCGCAGTG<br>- -
Wild type BGR1<br>*Δu2g07420DUF2169*<br>*Δu2g07420PPR*	GCCGCAGGCCAGCCTGCGCGGCGCCCGGCTGGGCCGCCTGCTCGGGCTGC<br>GCCGCAGGCCAGCCTGCGCGGCGCCCGGCTGGGCCGCCTGCTCGGGCTGC<br>- -
Wild type BGR1<br>*Δu2g07420DUF2169*<br>*Δu2g07420PPR*	GGCTCGCCTGCCCCGGCGCCGTGTTCGACGAGGCCGACGCGAGCAAGGCA<br>GGCTCGCCTGCCCCGGCGCCGTGTTCGACGAGGCCGACGCGAGCAAGGCA<br>- -
Wild type BGR1<br>*Δu2g07420DUF2169*<br>*Δu2g07420PPR*	ACGCTGTTCGACCTCGCGGCCGAGGACAGCCGCTGGCATGCCGCGCGGCT<br>ACGCTGTTCGACCTCGCGGCCGAGGACAGCCGCTGGCATGCCGCGCGGCT<br>- -
Wild type BGR1<br>*Δu2g07420DUF2169*<br>*Δu2g07420PPR*	CGAGAAGACGGTGTTCATGCGCGCGCGGCTGACGCGCGCCGGCTTCGCGG<br>CGAGAAGACGGTGTTCATGCGCGCGCGGCTGACGCGCGCCGGCTTCGCGG<br>- -
Wild type BGR1<br>*Δu2g07420DUF2169*<br>*Δu2g07420PPR*	GCGCGCGGCTGACCAAGACCGTGTTCACCATGAGCGACCTGCAGGCGAGC<br>GCGCGCGGCTGACCAAGACCGTGTTCACCATGAGCGACCTGCAGGCGAGC<br>- -
Wild type BGR1<br>*Δu2g07420DUF2169*<br>*Δu2g07420PPR*	CGCTGGGAAGGCGCGCGGCTCGACGGCGTGCAGGGCGGCACCCGGAGCAT<br>CGCTGGGAAGGCGCGCGGCTCGACGGCGTGCAGGGCGGCACCCGGAGCAT<br>- -
Wild type BGR1<br>*Δu2g07420DUF2169*<br>*Δu2g07420PPR*	CTGGCGCGACGCCGTGATGAGCGGCGTGACGGCGCGCAACAGCGGCTTCG<br>CTGGCGCGACGCCGTGATGAGCGGCGTGACGGCGCGCAACAGCGGCTTCG<br>- -
Wild type BGR1<br>*Δu2g07420DUF2169*<br>*Δu2g07420PPR*	CCGGCGCCGACCTCTCGCACGCCGATCTGGAGGCGGCCAGCTTCCTGCGC<br>CCGGCGCCGACCTCTCGCACGCCGATCTGGAGGCGGCCAGCTTCCTGCGC<br>- -
Wild type BGR1<br>*Δu2g07420DUF2169*<br>*Δu2g07420PPR*	TGCGACTTCGGCCAGGCCGAGCTGCGCGGCGCCCGGCTGGTGGCCGGCCT<br>TGCGACTTCGGCCAGGCCGAGCTGCGCGGCGCCCGGCTGGTGGCCGGCCT<br>- -
Wild type BGR1<br>*Δu2g07420DUF2169*<br>*Δu2g07420PPR*	GTTCTCGCACTGCGGCTTCTATGCGAGCCGGCTGCGCATGGTCGAGGCAA<br>GTTCTCGCACTGCGGCTTCTATGCGAGCCGGCTGCGCATGGTCGAGGCAA<br>- -
Wild type BGR1<br>*Δu2g07420DUF2169*<br>*Δu2g07420PPR*	GCGGCGCCGAGTTCTACCAATGCACCTGCCGCAAGACCGATTTCACCGGC<br>GCGGCGCCGAGTTCTACCAATGCACCTGCCGCAAGACCGATTTCACCGGC<br>- -
Wild type BGR1<br>*Δu2g07420DUF2169*<br>*Δu2g07420PPR*	GCGCGGCTGGTGGACGCCGTGTTCGCGCAGTGCGAGCAGACCGGCGCGAT<br>GCGCGGCTGGTGGACGCCGTGTTCGCGCAGTGCGAGCAGACCGGCGCGAT<br>- -
Wild type BGR1<br>*Δu2g07420DUF2169*<br>*Δu2g07420PPR*	CCCGCCGCAAGGCGTCACCACCGGGAGCCCCGCATGA<br>CCCGCCGCAAGGCGTCACCACCGGGAGCCCCGCATGA<br>----------------------------------TGA

Supplementary Table S4. Oligonucleotide primers used in this study.

Name	Sequence (5'→3')	Use
*u2g07410*_LF	AAAAAGCTTGCGCCGAGTTCTACCAAT	To amplify the L fragment of *bglu_2g07410*
*u2g07410*_LR	CGTCGCGTTATGAGAAGTCCACCTCGCT	To amplify the L fragment of *bglu_2g07410*
*u2g07410*_RF	ATCACGCCCAAATGCATAA	To amplify the R fragment of *bglu_2g07410*
*u2g07410*_RR	TTTTGAATTCGAGCAGATAGGCGTGCTGT	To amplify the R fragment of *bglu_2g07410*
*u2g07410*_UP_F	ACCAAGACCGTGTTCACCAT	To confirm the disruption of *bglu_2g07410*
*u2g07410*_DOWN_R	ACAGCACGTGCAGGATGTAG	To confirm the disruption of *bglu_2g07410*
*u2g07420DUF2169*_LF	TTTTGAATTCGTCGAGGAAATGGACGTGA	To amplify the L fragment of the DUF2169 domain in *bglu_2g07420*
*u2g07420DUF2169*_LR	GCGGCGCGCCTGGCGCCGGCCATTGCTGGTTCTC	To amplify the L fragment of the DUF2169 domain in *bglu_2g07420*
*u2g07420DUF2169*_RF	GACGCGGTCCTGAAGATT	To amplify the R fragment of the DUF2169 domain in *bglu_2g07420*
*u2g07420DUF2169*_RR	TTTAAGCTTTGCTCATGCAGAGGTTGG	To amplify the R fragment of the DUF2169 domain in *bglu_2g07420*
*u2g07420DUF2169*_UP_F	AAATCATCAAACCCCAATCG	To confirm the disruption of the DUF2169 domain in *bglu_2g07420*
*u2g07420DUF2169*_DOWN_R	GCATAGCTGAGCGGCATC	To confirm the disruption of the DUF2169 domain in *bglu_2g07420*
*u2g07420PPR*_LF	AAAGGATCCAAGCTGCTGGCCGACGCG	To amplify the L fragment of the pentapeptide repeats region

		in *bglu_2g07420*
*u2g07420PPR*_LR	AGATCGTGCTCATTTGGCGGATCTGCTCGCC	To amplify the L fragment of the pentapeptide repeats region in *bglu_2g07420*
*u2g07420PPR*_RF	AAGGCGTCACCACCGGGAG	To amplify the R fragment of the pentapeptide repeats region in *bglu_2g07420*
*u2g07420PPR*_RR	TTTAAGCTTTTGACGAAGCTCGCGTGG	To amplify the R fragment of the pentapeptide repeats region in *bglu_2g07420*
*u2g07420PPR*_UP_F	GATCTGTCGGAGATCGTGCT	To confirm the disruption of the pentapeptide repeats region in *bglu_2g07420*
*u2g07420PPR*_DOWN_R	GTTGACGATCGAGCTGGTCT	To confirm the disruption of the pentapeptide repeats region in *bglu_2g07420*
*u1g23320*_LF	AAAGGATCCTTGCCGCCGGGCACGTCG	To amplify the L fragment of *bglu_1g23320*
*u1g23320*_LR	CAGGTCTGGTCCAGATCCACGCGCCACATAC	To amplify the L fragment of *bglu_1g23320*
*u1g23320*_RF	AAGGTCAATTTGCTGGACC	To amplify the R fragment of *bglu_1g23320*
*u1g23320*_RR	TTTAAGCTTAGTTGGGCCAACTGCGCGC	To amplify the R fragment of *bglu_1g23320*
*u1g23320*_UP_F	CTTTTCCAGACGCTGACCTT	To confirm the disruption of *bglu_1g23320*
*u1g23320*_DOWN_R	GGTTCGCTGCAGTTTCTTCC	To confirm the disruption of *bglu_1g23320*
pk18_DOWN_R	GTGAAGCTAGCTTATCGCCAT	To confirm the first crossover in the process of constructing the deletion mutant.
C*u2g07410*_F	TTTAAGCTTATGAGCACGATCTGGGAC	Amplifying the *bglu_2g07410* fragment to be cloned in

		pBBR1MCS2.
C*u2g07410*_R	TTTTGAATTCTCATTC GTTCTCCAGGGC	Amplifying the *bglu_2g07410* fragment to be cloned in pBBR1MCS2.
C*u2g07420*_F	TTTAAGCTTAATGAAA ATCATCAAACCC	Amplifying the *bglu_2g07420* fragment to be cloned in pBBR1MCS2.
C*u2g07420*_R	TTTTGAATTCTCATGC GGGGCTCCCGGT	Amplifying the *bglu_2g07420* fragment to be cloned in pBBR1MCS2.
pB_UP_F	GACTCACTATAGGGC GAATTG	To confirm inserts gene fragments in pBBR1MCS2
pB_DOWN_R	CACACAGGAAACAG CTATGAC	To confirm inserts gene fragments in pBBR1MCS2

* Underlined sequence in the primers indicates the restriction enzyme-targeted sequences.
